# Supplementary material for: A Noninvasive Method for Time-Lapse Imaging of Microbial Interactions and Colony Dynamics
Source: Microbiol Spectr. 2022 Jul 5;10(4):e00939-22. doi: 10.1128/spectrum.00939-22 (PMC9430563; doi:10.1128/spectrum.00939-22)
Supplement: Supplemental file 1 — Supplemental material. Download spectrum.00939-22-s0001.pdf, PDF file, 0.5 MB [file spectrum.00939-22-s0001.pdf]

Title: A non-invasive method for time-lapse imaging of microbial interactions and colony dynamics

Authors: Carlos Molina-Santiago[1,*], John R. Pearson[2,*], María Victoria Berlanga-Clavero[1], Alicia Isabel Pérez-Lorente[1], Antonio de Vicente[1] and Diego Romero[1,#]

Supplementary Material

Supplementary Figures

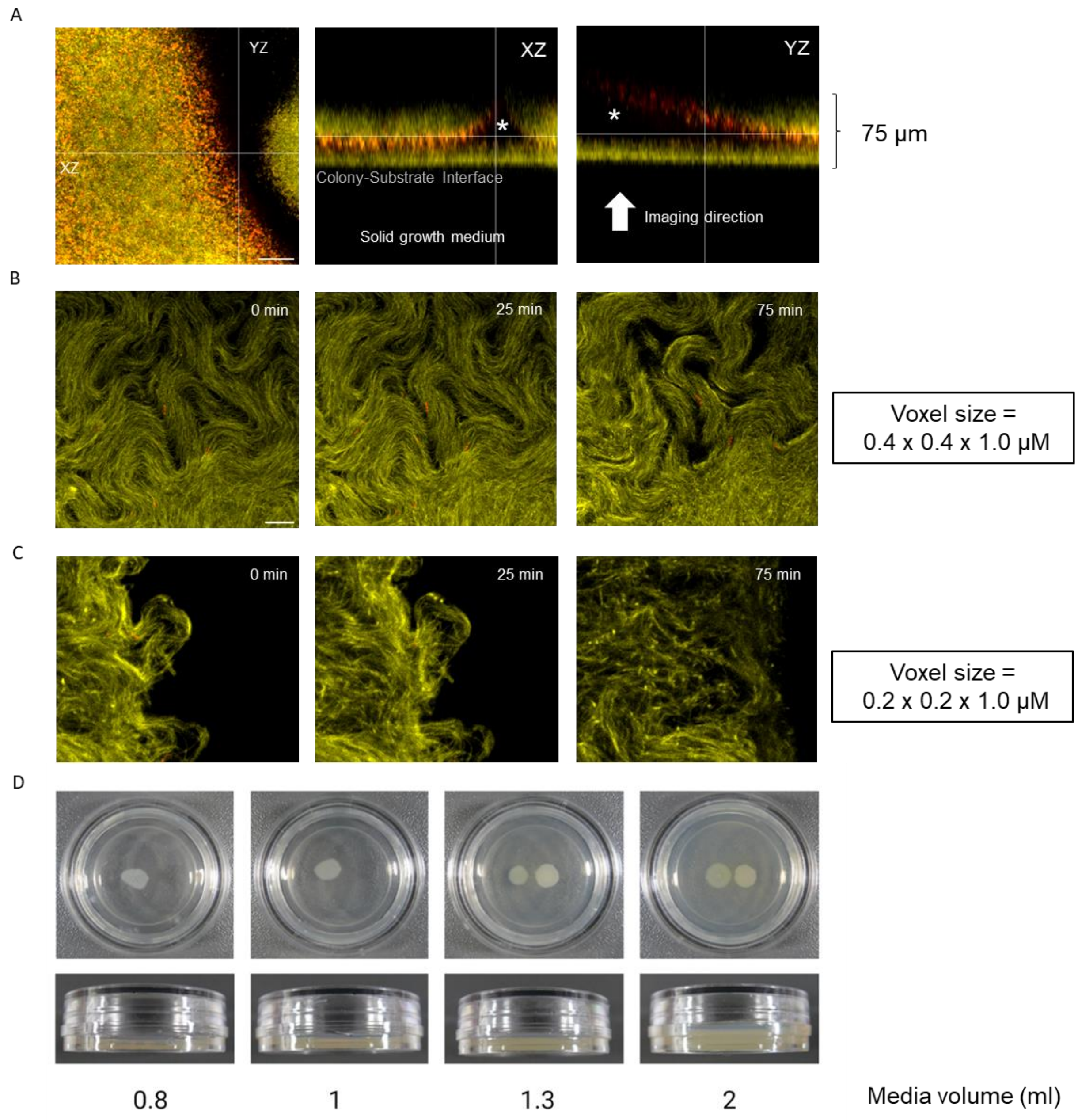

Supplementary Figure 1.A) Orthogonal views of a single time-point of the time-lapse showing B. subtilis promoter expression. Images illustrate the capacity of the BacLive method to visualize the 3D organization of fluorescent cells at a distance up to approximately

75 µm from the colony-substrate interface. Asterisks indicate regions with fewer bacteria or without fluorescent expression. B-C) Images showing a time-lapse (0, 25 and 75 min) of a B. subtilis colony expressing PmotA-YFP and PtasA-mcherry using the same 25x 0.95 NA water immersion objective lens using different acquisition conditions of view to obtain additional optical resolution at the cost of a smaller field of view and higher data sizes. B - Acquired using zoom x 1.5, 1024 x 1024 pixel format (shown cropped to 512 x 512) to achieve a voxel size of 0.4 x 0.4 x 1 µm. Scale bar = 25 µm. C - Acquired using zoom x 3, 1024 x 1024 pixel format (shown cropped to 512 x 512) to achieve a voxel size of 0.2 x 0.2 x 1 µm, close to the theoretical XY resolution limit of the objective (205 nm). Scale bar = 25 µm. D) Solid media volume and thickness is critical for the correct performance of the experiment. Glass bottomed Petri dishes with different culture media volumes show differences in bacterial growth. 1.3 ml of solid culture media was the best condition tested for the correct bacterial growth while allowing image acquisition.

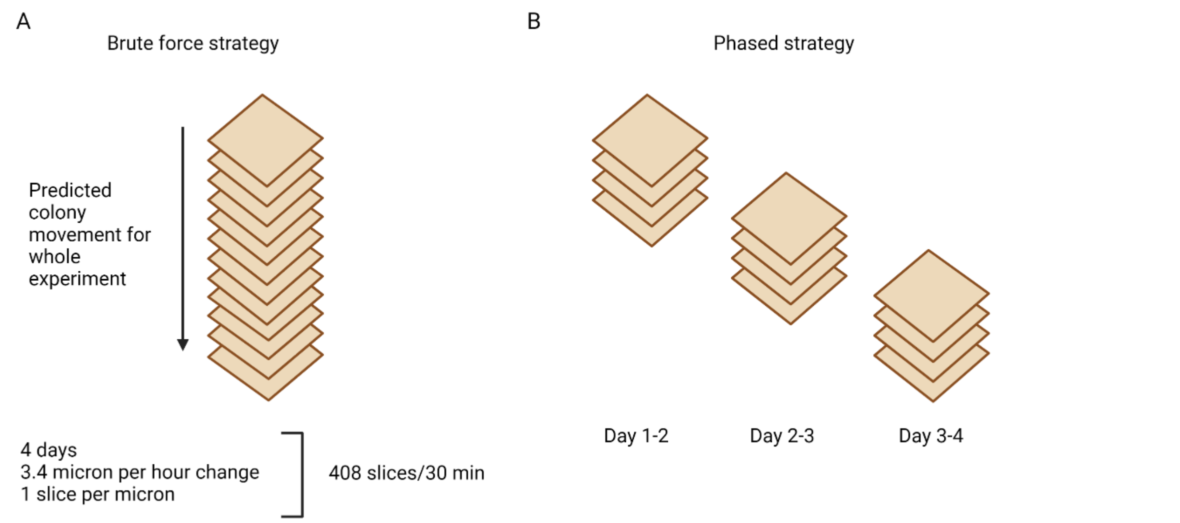

Supplementary Figure 2. Comparison of strategies for z-slices calculation and acquisition. A) Brute force strategy is based on capturing all the 3D volume where colonies are expected along the experiment. B) Phased strategy is based on the partial acquisition of 3D volumes according at different stages of the experiment.

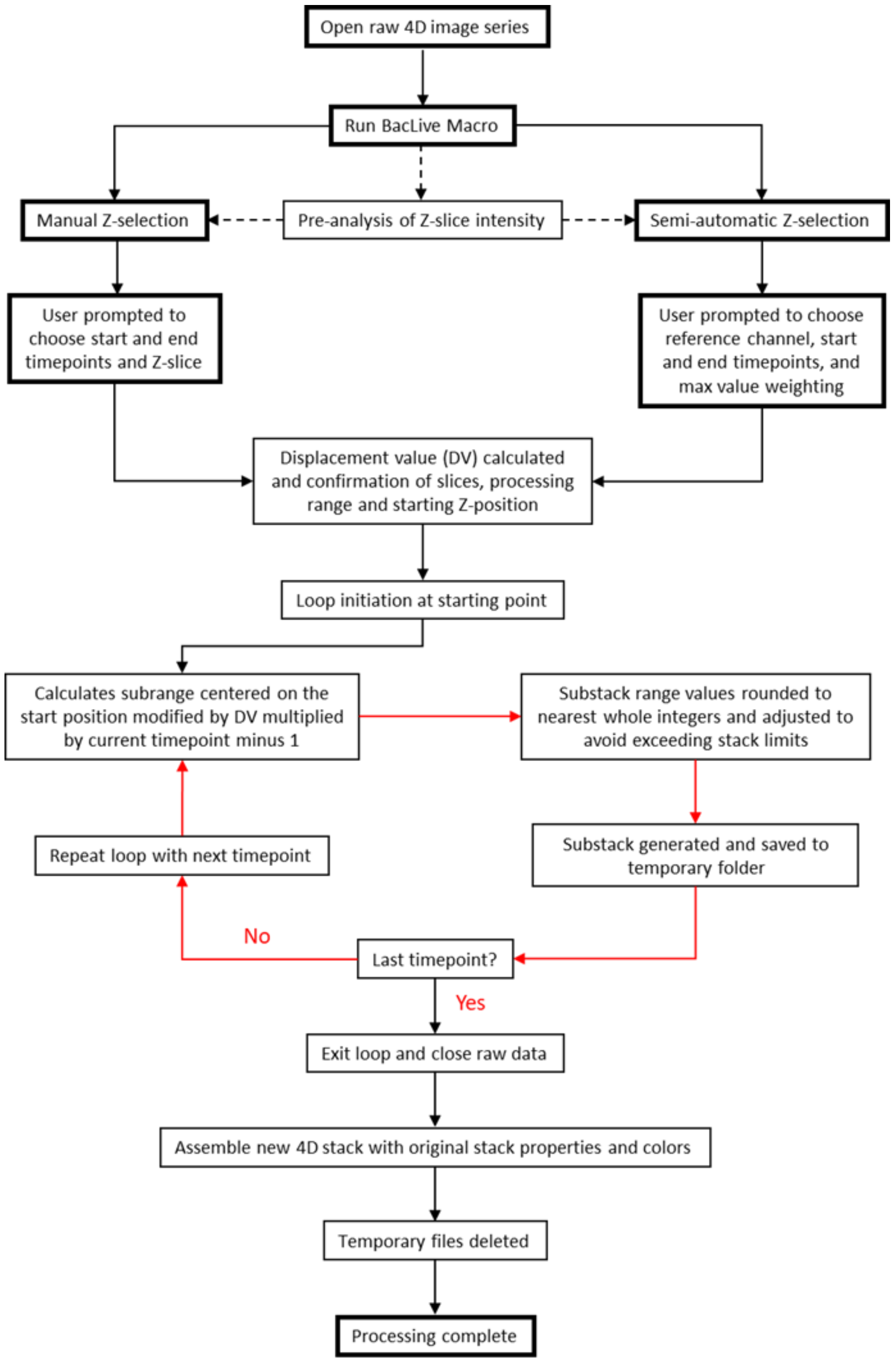

Supplementary Figure 3. BacLive workflow showing the steps performed for the processing of data acquired. Steps with a bold frame are key steps in the BacLive process. Red frames indicate steps repeated in the processing loop.

A

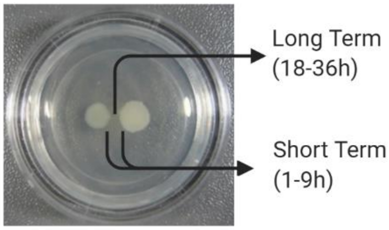

B

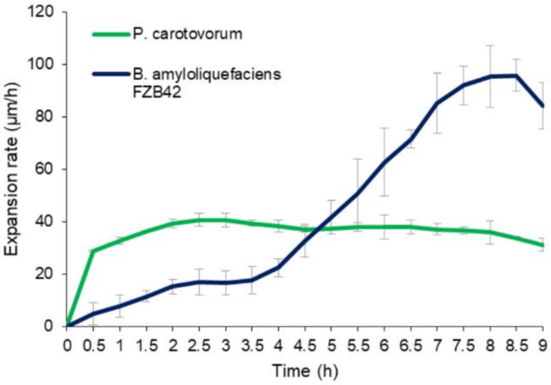

Supplementary Figure 4. A) Pairwise interaction representation indicating the exact spots used to analyze short-term and long-term stages of the interaction. B) Expansion rates of the FZB42 and *P. carotovorum* leading edges in the short-term stage of the interaction (9 h). The blue line represents the FZB42 leading edge, and the green line represents *P. carotovorum* leading edge. Error bars indicate SD. n = 3.

Supplementary Movies

Supplementary Movie 1. Long term 2D time lapse (18 – 36 h) of the interaction between *P. carotovorum* (green) and FZB42 (cyan).

Supplementary Movie 2. Long term 3D time lapse (18 – 36 h) of the interaction between *P. carotovorum* (green) and FZB42 (cyan). Bacterial colonies are represented as volumes.

Supplementary Movie 3. Long term 3D time lapse (18 – 36 h) of the interaction between *P. carotovorum* (green) and FZB42 (cyan).

Supplementary Movie 4. 3D time lapse of a *B. subtilis* colony leading edge. Spots indicate fluorescent bacteria expressing TasA (red) and MotA (yellow).

Supplementary Movie 5. 3D time lapse of a region of a *B. subtilis* colony where wrinkles are formed. Subpopulations expressing TasA (red) and MotA (yellow) are shown as volumes.

Supplementary Movie 6. 3D time lapse of a region of a *B. subtilis* colony where wrinkles are formed. Subpopulations expressing TasA (red) and MotA (yellow) are shown as volumes. Grey area indicates a region with cells not expressing TasA or MotA.
